# Psychosis and Gender: A Focus on Women in the Global South

**DOI:** 10.1177/07067437241295301

**Published:** 2024-11-03

**Authors:** Sarah Barber, Adiyam Mulushoa, Charlotte Hanlon, Ashok Malla

**Affiliations:** 1 Health Service and Population Research Department, Institute of Psychiatry, King’s College London, Psychology and Neuroscience, London, UK; 2 Department of Psychiatry, School of Medicine, 247395 College of Health Sciences, Addis Ababa University, Addis Ababa, Ethiopia; 3 Division of Psychiatry, 215746 Centre for Clinical Brain Sciences, University of Edinburgh, Edinburgh, Scotland, UK; 4 Department of Psychiatry, 5620 McGill University, Montreal, Quebec, Canada

**Keywords:** schizophrenia spectrum and other psychotic disorders, severe mental disorder, mental health services, women's health services, women's rights, Africa, Asia, Oceania, Latin America, low- and middle-income countries

## Introduction

Sex or gender differences are described as one of the most consistently reported features of schizophrenia. Studies—dominated by research from “the Global North” (largely North America and Europe)—tend to show that women have lower incidence, later onset and better clinical and functional course and outcome of illness than men.^1,2^ Purely biological explanations for this difference have been proposed, such as the protective effect of oestrogen.^3^

However, the axiomatic view that schizophrenia follows a more benign course in women has been challenged. Finnish registry data has recently demonstrated a higher rate of psychiatric rehospitalization in women with schizophrenia compared to men, as well as higher rates of suicide attempts and self-harm.^4^ In addition, some previous findings of a female “advantage” have been based on select samples, such as randomized clinical trials (RCTs).^5^ In other cases, varying lengths of treatment may confound the apparent difference.^6^ Non-RCT, large sample studies of first-episode psychosis patients show somewhat different results. One such study showed better clinical outcomes in women at 1 year, but similar clinical and functional outcomes at 2 years, suggesting men may take longer than women to achieve desired outcomes.^7^ This study also highlighted how reported sex differences in outcome may be at least partially attributable to risk factors that are unlikely to be purely biological, such as substance use and adherence to treatment.

Evidence of regional variation further points to gender-determined environmental factors being more salient in shaping the course of illness, rather than purely sex-determined biological factors.^8^ The Worldwide Schizophrenia Outpatient Health Outcomes study found that patterns of sex differences in remission and recovery between regions were mixed. Overall there was a marginal female advantage in recovery at 3 years (17% vs. 12%).^9^ However, the ability to draw strong conclusions from this facility-based study is limited by the risk of selection bias and underrepresentation of participants from the Global South, which broadly refers to the regions of Latin America, Asia (excluding Japan, Singapore, South Korea, and Taiwan), Africa and Oceania (excluding Australia and New Zealand), where the majority of the world's population lives.

The emerging consensus is that whilst men and women may differ in the nature of their risks for schizophrenia, the course of illness and their care needs, this is due to both biological and socio-cultural factors.^10^ As a result, findings from -one setting are unlikely to generalize to another. In this report, therefore, we highlight research on the impact of psychosis on the lives of women in the Global South. These settings are underrepresented in psychosis research, yet intersecting disadvantages for women, such as culturally enshrined role expectations, poverty and constrained treatment options may be more acute. Looking at outcomes that matter for women, we focus on domains of female empowerment over clinical indicators and where relevant, we highlight how findings deviate from global averages or challenge the axiomatic view of female advantage. However, we do not seek to make generalizations across the diverse socio-cultural contexts of the Global South. Instead, we draw heavily from the literature in settings best known to the authors, including Ethiopia and India. We also give examples of programmes seeking to provide culturally sensitive care to women with psychosis.

## Outcomes and Experiences of Women With Psychosis in the Global South

### Life and Good Health

Globally schizophrenia is associated with on average 14.5 years of potential life lost, slightly higher for men (15.9 years), than women (13.6 years).^11^ However, findings from Ethiopia are markedly different; in a rural cohort, the years of potential life lost were not only much higher overall, but higher in women (30.0 years) than men (26.9 years).^12^ Whilst suicide is an important contributor to excess mortality across settings, infection and malnutrition were the leading causes of death in Ethiopia,^12^ as compared to cardiovascular disease in higher-income settings.^13^

Mortality rates alone do not capture a broad view of life and good health. Sexual and reproductive health issues are especially relevant to women due to biological factors and socially defined gender roles.^14^ Women with schizophrenia in Turkey are disadvantaged, for example, they have higher rates of unplanned pregnancy and receive less antenatal care than women without schizophrenia.^15^ This pattern is not unique to Turkey or the Global South.^16^ However, context-specific provider-related factors may be important to understand this finding. Research from the Central Anatolia region of Turkey found that a significant proportion of nurses felt education around family planning for women with schizophrenia should be reserved for married individuals or not offered at all.^17^ In this region, prevailing traditional values and stigma may conspire to impede reproductive choice for women with schizophrenia.

### Relationships, Family Life, and Freedom From Violence

In research on the course and outcome of psychosis, it is often implicitly assumed that “marriage” is an indicator of individual functioning and “marriage rates” reflective of the socio-cultural environment for recovery.^18^ In a rural Chinese cohort study, more women than men with schizophrenia were indeed married at baseline and 14-year follow-up, however, this showed no association with functioning scores or ability to work.^19^

“Ever-married” rates also fail to give the full picture. Women with severe mental illness (SMI) (a term often used to signify the presence of psychosis) in India and Ethiopia are more likely to be abandoned, separated or divorced by their spouses than men^20,21^ often due to failure to fulfil expected domestic roles.^22,23^ In India, marriage and marriageability are regarded as among the most highly desirable achievements for women, offering security, social status and dignity.^22^ Marriage breakdown can lead to despair and even suicidality for women with SMI in India, although for some the loss was mitigated by the support and protection offered by their own parents and family.^22^

Whilst marriage can offer a source of stability for women, intimate partner violence (IPV) is the most common form of violence worldwide.^24^ Less empowered women have a greater risk of IPV^25^ and there is a bidirectional relationship between IPV and mental health.^24^ Some women with SMI may be particularly vulnerable to IPV due to the disorganizing effect of their illness and social stigmatization, but research data are lacking across all settings.^26^ In a study of female outpatients with schizophrenia in Nigeria, 75% reported a history of IPV and 25% reported previous sexual assault.^27^ Importantly, victims of IPV had significantly higher psychiatric symptom severity scores, demonstrating the compounding effect of mental illness and violent victimization. In qualitative studies in Ethiopia, women with SMI have disclosed nonpartner sexual assault, leading to unwanted pregnancies and further stigma; “They say ‘You are mentally ill and you give birth to a bastard?’”^28^ In addition, a study of street homeless people in Addis Ababa found that 3 out of 9 women with psychosis reported a history of sexual abuse.^29^ There are significant barriers to women in Ethiopia accessing reproductive health services, including safe abortion, and justice.

### Participation in Decision-Making

In some Global South settings, rigidly defined gender roles encoded in religious and social traditions can act to limit women's ability to make decisions about aspects of their own lives, including their health.^30,31^ Research exploring the family planning experiences and preferences of women with psychosis is scarce. One study in India found just 14% of women with schizophrenia had an unmet need for family planning.^32^ However, a closer inspection of the findings shows that informed choice regarding contraception was strikingly low. The majority of women were excluded from decisions about their contraception, which were instead made by their husbands, mother-in-law, other relatives, or doctors.^32^ Out of 65 women using contraception, 86% had undergone permanent sterilization.^32^ Exclusion from such a decision is highly concerning.

Exclusion of women from household decision-making may also lead to the prioritization of men's health needs, particularly in families with limited financial resources. This has been suggested as a plausible explanation for the longer duration of untreated psychosis (DUP) in women with schizophrenia in India,^33^ albeit based on a small sample.^34^ Whilst global averages have not indicated a gender difference in DUP, this obscures the variability between settings and the highly contextual influencing factors,^35^ in addition to differences in measurement.

### Education, Skill-Building and Knowledge, Labour and Financial Inclusion

Employment status is considered an important measure of functioning and social reintegration in schizophrenia. Like marriage, higher rates of employment in “developing countries” were considered to indicate better outcomes in early cross-cultural comparative studies of schizophrenia.^36^ However, consideration of the quality of employment and drivers such as lack of social security has led to a reframing of employment as another crude and potentially misleading indicator.^37^

Around the world, women spend more time engaged in unpaid and domestic work and are overrepresented in the informal or casual labour market with minimal protection. There is little research on the educational and vocational attainment or needs of women with psychosis in the Global South. In keeping with general trends, rates of paid employment are higher in men than women with schizophrenia in East Africa, North Africa and the Middle East.^38^ Yet there is evidence of aspiration, with over 70% of women with SMI in India desiring employment.^39^

## Intervening to Support Recovery

Supporting recovery in women with schizophrenia requires a multisectoral response that is centred on women's needs and preferences, draws on community resources^40^ and is adapted to socio-cultural context.

### Integrated Mental Health Care Services

Most people with schizophrenia in the Global South do not have access to mental health services; the estimated treatment gap in low-income countries is over 90% and gender-disaggregated data are lacking. Efforts to expand mental health services have focused on the integration of mental health into primary and, for women, maternal care platforms. In this model, general nurses, midwives and mid-level nonphysician health professionals are trained and supervised to deliver evidence-based mental health care, including prescription of antipsychotic medications.^41^ The Programme for Improving Mental Health Care (PRIME) in rural Ethiopia demonstrated that both men and women with SMI (256 out of 300 (85.3%) of whom had schizophrenia) benefited from integrated primary mental health care, with no gender difference in access or clinical, social or economic outcomes.^42,43^ This care model was also shown to reduce household food insecurity.^44^ Similar benefits for women with psychosis were seen in an RCT of integrated primary mental health care in Ethiopia.^45^ Physical health needs may also be better served by this integrated approach,^45^ although there is limited evidence on interventions to improve physical health and reduce mortality in women with schizophrenia.

In some settings where the movement of women is constrained, for geographical or cultural reasons, women may not readily access facility-based health care. Near-home and home-based models of mental health care by community health workers have been implemented for perinatal common mental disorders in Pakistan with some promising results,^46^ however, this model has not been evaluated in relation to women with schizophrenia.

### Specialist Perinatal Mental Health Care Services

Recent guidelines for integrated perinatal mental health and maternal and child health services suggest that women with severe mental health conditions, including psychosis, require more intensive interventions that are delivered or supervised by specialists.^47^ This is due to complexities related to prescribing in pregnant and breastfeeding women, as well as their risk of adverse obstetric outcomes and psychiatric outcomes.^48^ There are examples of excellent specialist models of care in tertiary referral settings, for example, the Mother and Baby Unit in Bengaluru, India.^49^ However reliance on specialists creates a barrier to accessing services in many settings, concentrated in the Global South, where there is less than one mental health professional per 100,000 of the population.^50^

Where specialist care is needed, innovative solutions will be required to reach underserved populations, with community outreach and digital platforms already showing potential in mental health care generally.^51,52^ However, improving access to universal sexual and reproductive services for women with psychosis should remain a priority.

### Responding to Gender-Based Violence

All contacts with health services are an opportunity to recognize and respond to violence against women,^53^ including women with psychosis. Interprofessional, health-system-based models of care such as “One Stop Centres” have emerged in Africa and Asia, aiming to provide comprehensive services for women, including medical, legal and psychosocial support.^54^ However various factors have hindered implementation of the model and the achievement of intended results. For women with psychosis, stigma and discrimination likely create additional barriers to accessing and benefiting from such services.^55^ There is also a need for interventions to protect women from violence occurring within inpatient mental health settings.

### Economic Interventions

There is evidence that employment interventions for people (including women) with psychosis improve mental health outcomes and functioning and reduce social disability in people with SMI both in China^56^ and India,^57^ in line with findings from the Global North.^58^ This is an area that requires further investment in research and development. Not only is participation in economic life an important part of social inclusion for people with psychosis, but an extensive body of research shows that women's economic independence has far-reaching benefits, not least a reduction in gender-based violence.^59^

## Conclusion

Close interrogation of data across more diverse settings challenges the assumption of a universal female advantage in the course and outcome of psychosis. Research in underrepresented settings such as Ethiopia and India highlights the complex interplay of biological, social, and cultural factors in shaping outcomes. In these settings, we have reported that women with psychosis face high mortality rates, limited access to health services and high levels of gender-based violence. Stigma relating to mental illness, socio-cultural gender norms and economic constraints act to exacerbate these challenges.

Efforts to support recovery and improve outcomes for women with psychosis require a multisectoral response. Whilst this may involve specialists, in resource-constrained environments (concentrated in the Global South), approaches that rely on parallel or siloed systems are likely to be inaccessible to the majority of the population. Reducing barriers to accessing universal and integrated services will be crucial, as well as focusing on interventions to improve the social inclusion of women with psychosis, such as participation in economic life. As research efforts continue, it is vital to tailor interventions to respond to the specific needs of women in different socio-cultural contexts.
